# Cardiac Circular RNAs CDR1as, Circ-RCAN2, Circ-C12orf29 Show Cell-Specific Hypoxia-Induced Dysregulation and Distinct In Vitro Effects

**DOI:** 10.3390/ijms262110334

**Published:** 2025-10-23

**Authors:** Ena Hasimbegovic, Dominika Lukovic, Nina Kastner, Benedikt S. Hofer, Andreas Spannbauer, Denise Traxler, Julia Mester-Tonczar, Kevin Hamzaraj, Emilie Han, Martin Riesenhuber, Babette Maleiner, Katrin Müller-Zlabinger, Mariann Gyöngyösi

**Affiliations:** 1 Division of Cardiology, Department of Internal Medicine II, Medical University of Vienna, 1090 Vienna, Austria; 2 Institute of Pharmacology, Center of Physiology and Pharmacology, Medical University of Vienna, 1090 Vienna, Austria; 3 Division of Gastroenterology and Hepatology, Department of Internal Medicine III, Medical University of Vienna, 1090 Vienna, Austria; 4 Department of Oral and Maxillofacial Surgery, Medical University of Vienna, 1090 Vienna, Austria; 5 Department of Transfusion Medicine and Cell Therapy, Medical University of Vienna, 1090 Vienna, Austria

**Keywords:** circular RNA, hypoxia, small interfering RNA, myocardial infarction

## Abstract

Circular RNAs (circRNAs) are looped RNA molecules with regulatory roles in myocardial infarction and post-infarction cascades. We aimed to (i) confirm the circularity of novel circRNAs (CDR1as, circ-RCAN2, circ-C12orf29) implicated in myocardial infarction, (ii) examine cell-specific regulation patterns under hypoxia, and (iii) assess their effects on cell viability and downstream miRNA targets. Experiments were conducted on porcine cardiac progenitor cells (pCPCs), bone marrow mesenchymal stem cells (pMSCs) and cardiac fibroblasts (pCFs). Circularity was assessed by RNase R treatment, subsequent qPCR, gel electrophoresis and Sanger sequencing. Hypoxia experiments with/without serum deprivation mimicked ischemia. Effects on viability with/without hypoxia (MTT assay) and downstream miRNA targets were assessed via short interfering RNA (siRNA)-mediated knockdown of circ-RCAN2 and circ-C12orf29. Following RNase R treatment, qPCR product electrophoresis demonstrated amplification of singular products for all circRNAs, with backsplice junction amplification confirmed via Sanger sequencing. Serum deprivation and hypoxia resulted in cell-specific circRNA expression patterns, with an upregulation of all candidates in pCPCs across all intervals of hypoxia, an upregulation of circ-RCAN2 and circ-C12orf29 in pMSCs with prolonged hypoxia, and no detectable dysregulation in pCFs. siRNA knockdown of circ-RCAN2 reduced pCF- and increased pMSC-viability. circ-C12orf29 knockdown increased pCPC- and reduced pMSC-viability. circ-C12orf29 knockdown also upregulated ssc-miR-21-5p and ssc-miR-181c in pCPCs, with no detectable targets for circ-RCAN2. In conclusion, CDR1as, circ-RCAN2 and circ-C12orf29 are circular and dysregulated in a time- and cell-type-specific manner following hypoxia. circ-RCAN2 and circ-C12orf29 exhibit cell-type specific effects on viability, with circ-C12orf29 also targeting downstream miRNAs.

## 1. Introduction

Ischemic heart disease, and myocardial infarction as its acute manifestation, are significant contributors to global cardiovascular mortality [1]. Currently available treatments for myocardial infarction primarily focus on the rapid restoration of myocardial perfusion [2]. However, adverse myocardial remodeling frequently occurs following the event, resulting in impaired myocardial function and progressive heart failure [3]. Understanding the complex ensuing dysregulation following myocardial infarction on all levels is key to developing targeted treatment approaches. Circular RNAs (circRNAs), a class of looped covalently closed RNA molecules initially discovered in 1976, whose abundance and regulatory roles have been gaining increasing recognition during the previous decade, are a comparatively novel addition to this field of research [4,5,6]. Studies have already identified numerous promising circRNA candidates involved in post-infarction regulatory cascades, as well as their downstream targets [7,8,9,10]. The multi-layer effects of circRNAs in this context are perhaps best illustrated by circRNA Nfix, which showed cell type- and stage-specific expression [7]. An in vitro knockdown of circRNA Nfix induced cardiomyocyte proliferation, whereas an in vivo knockdown inhibited post-infarction cardiomyocyte death in mice [7]. Both protein and miRNA downstream targets, including interaction with Y-box binding protein 1 protein and microRNA (miRNA) miR-214, appear to play a role in conveying these effects [7]. Interestingly, the same circRNA was downregulated in hypertrophic cardiomyocytes and mice hearts [11]. When upregulated, it appeared to have a beneficial effect on the progression of cardiac hypertrophy in an animal pressure overload model, and hinder cardiomyocyte hypertrophy in vitro. In this study, Nfix appeared to target miR-145-5p, thus highlighting the context dependent roles of circRNA [11]. Other candidate circRNAs, such as MICRA, might even be able to predict impaired left ventricular function following myocardial infarction or play a role in determining post-infarction remodeling [12,13].

Two studies conducted by our research group on porcine large animal models of reperfused acute myocardial infarction uncovered a number of differentially regulated circRNAs and confirmed the expression of CDR1as in porcine hearts using qPCR and Sanger sequencing [14,15]. Herein, increased CDR1as expression was linked to improved ventricular function and reduced infarct size at two months following myocardial infarction, yet no significant difference in CDR1as expression between the infarcted and non-infarcted regions could be established [14]. CDR1as, which was among the first circRNAs to be characterized in animals, has been implicated in both cardiovascular and non-cardiovascular pathologies [16,17]. Mechanistically, CDR1as has multiple highly conserved binding sites for miR-7 [16]. It has been shown to be upregulated in murine myocardial infarction and hypoxic mouse cardiomyocytes, increase post-infarction myocardial injury and its knockdown could potentially alleviate the changes leading to myocardial infarction-related arrhythmias in mice [9,18]. Increased circulating levels of CDR1as have also been detected in patients with myocardial infarction [19]. Further effects attributed to CDR1as, potentially conveyed via other downstream targets, include the promotion of insulin secretion in islet cells upon overexpression of CDR1as, a potential role in cardiomyocyte apoptosis in diabetic cardiomyopathy, as well as the modulation of migratory and proliferative effects in cancer and non-cancer cells [20,21,22,23].

In addition to CDR1as, a 2021 study on porcine myocardium sampled on the third day following a reperfused myocardial infarction, our group uncovered a number of circRNAs with differential expression profiles in infarcted versus healthy myocardium, amongst them circ-RCAN2 and circ-C12orf29 [15]. However, further mechanistic studies have not yet been conducted on these novel candidates. Consequently, despite the apparent significant dysregulation in infarcted tissue, not much is known regarding the precise patterns of dysregulation under hypoxia, or the downstream effects and targets of circ-RCAN2 and circ-C12orf29.

Thus, the aims of this paper were to (i) confirm the circular nature of these novel transcripts, (ii) examine the differential regulation of their expression across a wide range of hypoxia intervals in vitro in porcine cardiac progenitor cells (pCPCs) and bone marrow mesenchymal stem cells (pMSCs), both of which have supportive regenerative potential following myocardial infarction and cardiac fibroblasts (pCFs), which play a role in post-infarct remodeling, and (iii) observe their potential effects on cell viability and downstream miRNA targets.

## 2. Results

### 2.1. Cell Characterization

**Figure 1:**
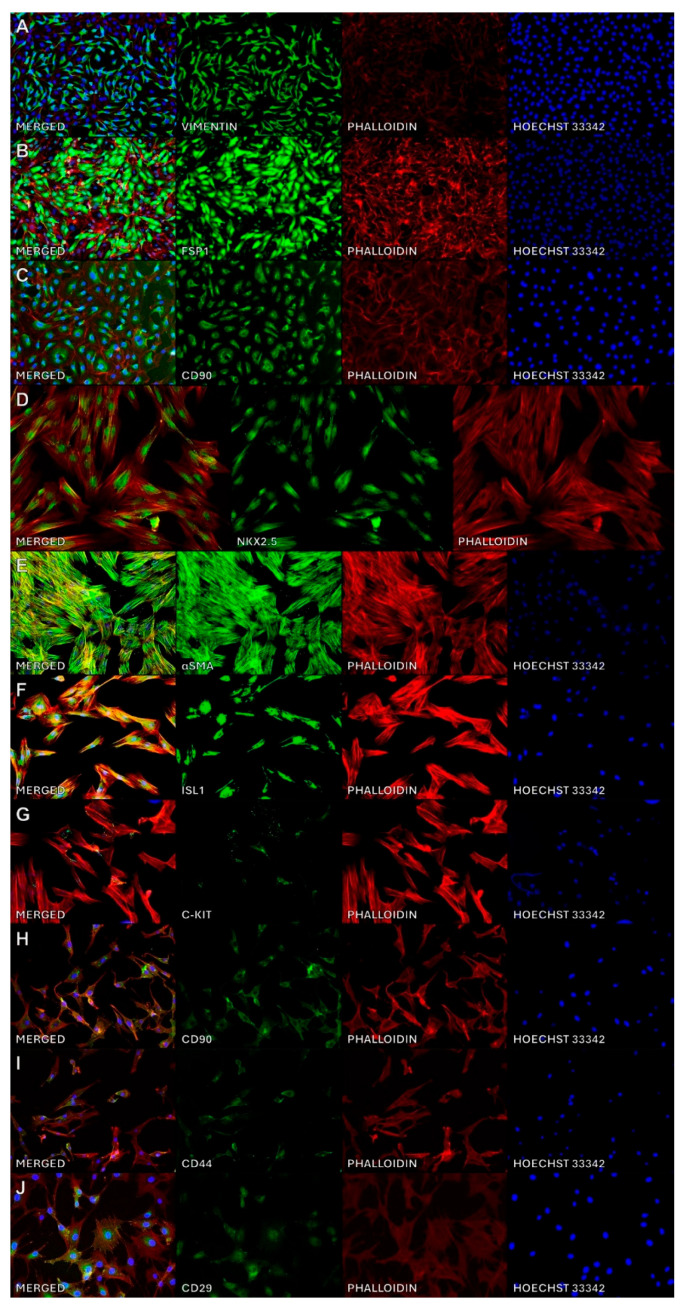
Immunofluorescence characterization of pCFs (**A**–**C**), pCPCs (**D**–**G**) and MSCs (**H**–**J**). Each row depicts the individual and merged channels for each target, with the merged image on the left. Abbreviations: porcine cardiac progenitor cells (pCPCs), porcine mesenchymal stem cells (pMSCs), porcine cardiac fibroblasts (pCFs), S100A4/Fibroblast-specific protein 1 (FSP1), NK2 Homeobox 5 (NKX2.5), Alpha smooth muscle Actin (αSMA), Islet-1 (Isl-1).

The characterization of pCPCs, pMSCs and pCFs using immunofluorescence for antigens present in each of the cell lines is shown in Figure 1. pCPCs demonstrated a characteristic expression of NK2 homeobox 5 (Nkx2.5), alpha smooth muscle actin (αSMA), Islet-1 (Isl-1) and c-kit. In pMSCs, the presence of CD90, CD44 and CD29 was successfully demonstrated. pCFs showed a characteristic expression of Vimentin, S100A4/Fibroblast-specific protein 1 (FSP1) and CD90.

### 2.2. The Transcripts Are Circular and Amplified Specifically by the Designed Primers

**Figure 2:**
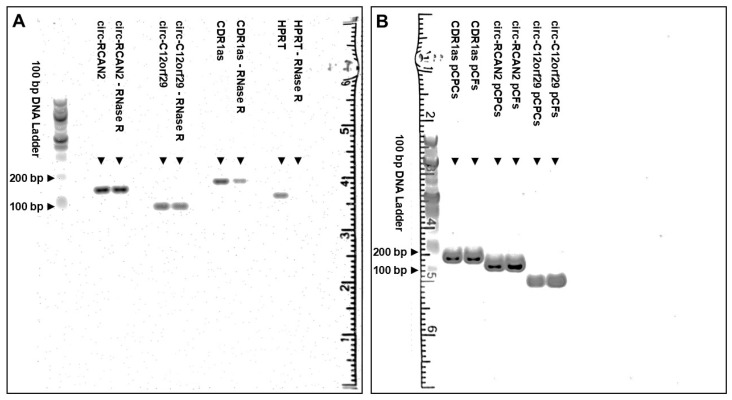
Agarose electrophoresis of PCR products. The left panel (**A**) illustrates agarose gel electrophoresis of qPCR products from pCPCs with and without RNase R treatment. The samples on the right panel (**B**) depict qPCR products from pCPCs and pCFs without RNase R treatment. The samples moved through the gel from top to bottom. HPRT was run as a linear treatment control. Abbreviations: porcine cardiac progenitor cells (pCPCs), porcine mesenchymal stem cells (pMSCs), porcine cardiac fibroblasts (pCFs), base pairs (bp).

**Figure 3:**
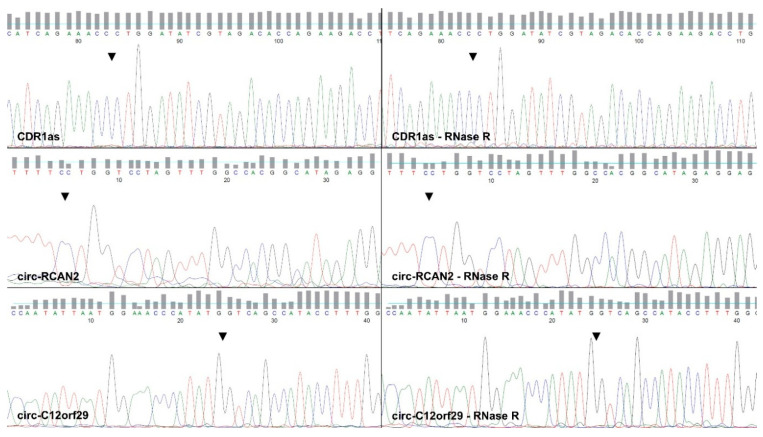
CDR1as, circ-RCAN2, circ-C12orf29 BSJ amplification with and without RNase R treatment confirmed by Sanger sequencing. The position of the respective BSJ is indicated by the arrow. The depicted traces stem from pCPC (undigested) and pMSC (RNase R) qPCR products. Abbreviations: porcine cardiac progenitor cells (pCPCs), porcine mesenchymal stem cells (pMSCs).

Agarose gel electrophoresis demonstrated that our selected primers for circ-RCAN2, circ-C12orf29 and CDR1as led to the amplification of singular qPCR products of the same lengths with and without RNase R treatment in RNA from pCPCs (Figure 2A), confirming the circular nature of the transcripts. While the band intensity was comparable for circ-RCAN2 and circ-C12orf29 regardless of RNase R treatment, there was some loss in intensity of the CDR1as lane after RNase R treatment. In contrast, linear RNA, as illustrated by HPRT, was completely degraded by RNase R treatment under the same conditions. As predicted during primer design, the product length of circ-C12orf29 (106 bp), circ-RCAN2 (163 bp) and CDR1as (193 bp) was reflected by the degree of migration of the amplified products relative to one another and the DNA ladder. The amplification of singular qPCR products of the same lengths in pCPCs and pCFs was confirmed in a second agarose gel (Figure 2B). The amplification of the backsplice junction (BSJ) was confirmed by Sanger sequencing of qPCR products from undigested and RNase R-treated RNA amplified by the designed primers as shown in Figure 3.

### 2.3. Hypoxia Chamber

**Figure 4:**
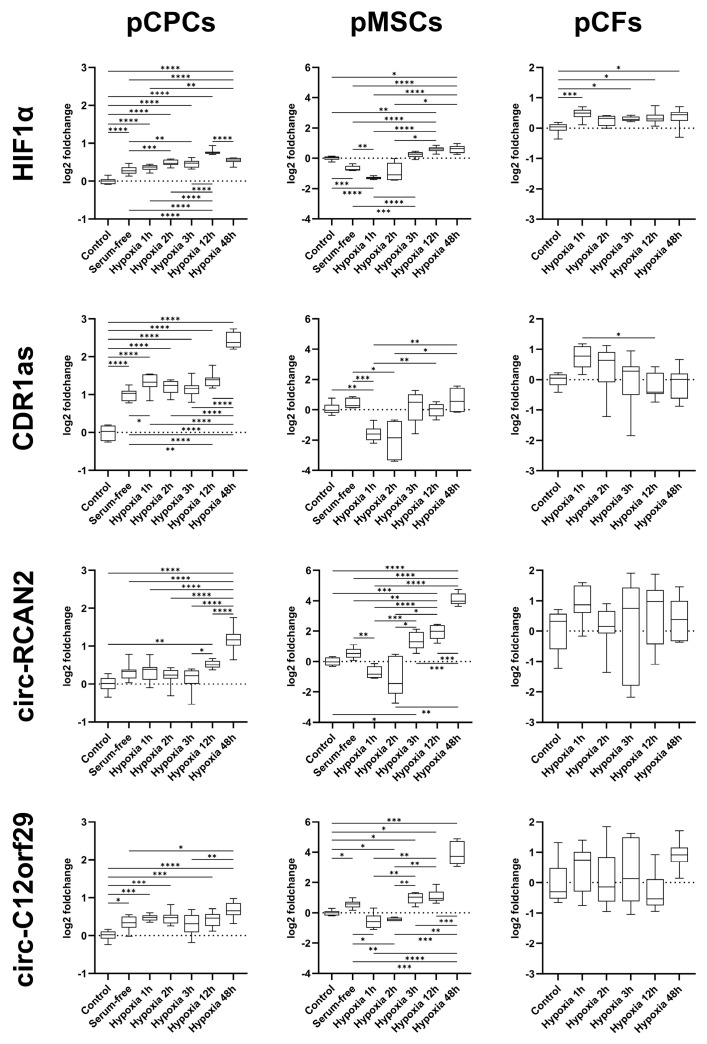
Expression of HIF1α, CDR1as, circ-RCAN2 and circ-C12orf29 after progressively longer intervals of hypoxia. Values are listed for pCPCs, pMSCs and pCFs. The change in expression is illustrated on a logarithmic scale to base 2 on a box and whiskers plot with the box representing the first and third quartile, the line in the plot representing the median and the whiskers representing the minimum and maximum values. The level of significance is illustrated as * *p* = 0.01–0.05, ** *p* = 0.001–0.01, *** *p* = 0.0001–0.001 and **** *p* < 0.0001. Abbreviations: porcine cardiac progenitor cells (pCPCs), porcine mesenchymal stem cells (pMSCs), porcine cardiac fibroblasts (pCFs).

In pCPCs, serum deprivation and hypoxia induced a significantly increased expression of hypoxia inducible factor 1 alpha (HIF1α) (Supplementary Table S1A and Figure 4). Specifically, the expression of HIF1α was increased by serum deprivation and at every interval of hypoxia with respect to the control cells (Tukey’s adjusted *p* < 0.001 for all). Hypoxia intervals of 2 h (Tukey’s adjusted *p* < 0.001), 3 h (Tukey’s adjusted *p* = 0.002), 12 h (Tukey’s adjusted *p* < 0.001) and 48 h (Tukey’s adjusted *p* < 0.001) additionally increased the expression of HIF1α relative to cells cultured in serum-free medium. The expression of HIF1α remained constant between 2 h and 3 h of hypoxia, increased between 3 and 12 h (Tukey’s adjusted *p* < 0.001) and decreased again between 12 and 48 h (Tukey’s adjusted *p* < 0.001). For CDR1as, serum starvation as well as all hypoxia times increased the expression significantly with respect to controls (Tukey’s adjusted *p* < 0.001 for all). Interestingly, although hypoxia increased the CDR1as expression compared to serum-free medium at 1 h, 12 h and 48 h (Tukey’s adjusted *p* = 0.046, *p* = 0.005 and *p* < 0.001, respectively), a further steep increase was observed between 12 and 48 (Tukey’s adjusted *p* < 0.001) hours of hypoxia. For circ-RCAN2, serum deprivation alone only induced a numerical upregulation with respect to controls, while prolonged hypoxia of 12 and 48 h significantly upregulated the expression (Tukey’s adjusted *p* = 0.002 and *p* < 0.001, respectively). For circ-C12orf29, both serum deprivation (Tukey’s adjusted *p* = 0.023) and hypoxia of 1 (Tukey’s adjusted *p* < 0.001), 2 (Tukey’s adjusted *p* < 0.001), 12 (Tukey’s adjusted *p* = 0.001) and 48 (Tukey’s adjusted *p* < 0.001) hours increased the expression relative to controls. However, only prolonged hypoxia of 48 h resulted in a significant increase compared to serum deprivation alone (Tukey’s adjusted *p* = 0.002).

In MSCs, serum deprivation led to a significant decrease in HIF1α expression with respect to control cells (Dunnett’s T3 adjusted *p* < 0.001), which became more pronounced at 1 h of hypoxia (Dunnett’s T3 adjusted *p* = 0.002 vs. serum-free) (Supplementary Table S1B and Figure 4). By 3 h of hypoxia, the HIF1α decrease had reversed into a significant increase with respect to serum-free medium (Dunnett’s T3 adjusted *p* < 0.001). At 12 and 48 h of hypoxia, HIF1α was consistently upregulated with respect to controls (Dunnett’s T3 adjusted *p* = 0.003 and *p* = 0.016, respectively). Importantly, CDR1as was not significantly differentially regulated by serum deprivation (Dunnett’s T3 adjusted *p* = 0.832), but at 1 h hypoxia, a downregulation with respect to both controls and serum-free cells could be observed (Dunnett’s T3 adjusted *p* = 0.003 and *p* < 0.001, respectively). A significant upregulation of CDR1as with respect to controls and serum-free cells could not be observed at any hypoxia interval. For circ-RCAN2, no significant up- or downregulation was induced by serum deprivation (Dunnett’s T3 adjusted *p* = 0.171). Hypoxia for 1 h resulted in a significant drop in circ-RCAN2 expression relative to serum-free medium (Dunnett’s T3 adjusted *p* = 0.003). From 3 h of hypoxia onwards, a significant increase in the expression of circ-RCAN2 with respect to controls (Dunnett’s T3 adjusted *p* = 0.019, *p* < 0.001 and *p* < 0.001 at 3 h, 12 h and 48 h, respectively) was observed, and from 12 h, expression levels were also increased with respect to serum-free medium (Dunnett’s T3 adjusted *p* < 0.001 and *p* < 0.001 at 12 h and 48 h, respectively). A further increase in expression was observed between 12 h and 48 h (Dunnett’s T3 adjusted *p* < 0.001). A similar expression pattern was present in circ-C12orf29, which increased significantly with serum deprivation (Dunnett’s T3 adjusted *p* = 0.032) and decreased after 1 h of hypoxia (Dunnett’s T3 adjusted *p* = 0.021). A significant increase in expression compared to controls could be observed after 2 h (Dunnett’s T3 adjusted *p* = 0.019), 3 h (Dunnett’s T3 adjusted *p* = 0.012), 12 h (Dunnett’s T3 adjusted *p* = 0.010) and 48 h (Dunnett’s T3 adjusted *p* < 0.001), with a pronounced increase between 12 h and 48 h (Dunnett’s T3 adjusted *p* < 0.001).

In pCFs, hypoxia induced a significantly increased expression of HIF-1α at 1 h (Tukey’s adjusted *p* < 0.001), 3 h (Tukey’s adjusted *p* = 0.045), 12 h (Tukey’s adjusted *p* = 0.015) and 48 h (Tukey’s adjusted *p* = 0.023) (Supplementary Table S1C and Figure 4), with no changes between the hypoxia intervals. Importantly, no significant differences were detected between non-hypoxic and hypoxic cells nor between different hypoxia points themselves for circ-RCAN2 or circ-C12orf29. CDR1as merely showed an increase after 1 h (Tukey’s adjusted *p* = 0.123), which dropped significantly with 12 h of hypoxia (Tukey’s adjusted *p* = 0.022).

### 2.4. siRNA-Induced Selective Downregulation of Circ-RCAN2 and Circ-C12orf29

Transfection of pCPCs with the siRNA for circ-RCAN2 led to a selective downregulation of the circular (−1.25 ± 0.14 vs. 0.00 ± 0.22 log2 FC; Tukey’s adjusted *p* < 0.001; Supplementary Figure S3) but not the linear variant compared to controls (0.33 ± 0.20 vs. 0.00 ± 0.18 log2 FC for controls). Similarly, the siRNA-targeting circ-C12orf29 lead to a significant downregulation of circ-C12orf29 compared to control cells (−1.77 ± 0.46 vs. 0.00 ± 0.24 log2 FC; Tukey’s adjusted *p* = 0.002), without affecting the expression of the linear variant (0.05 ± 0.11 vs. 0.00 ± 0.10 log2 FC).

In pMSCs, the siRNA-targeting circ-RCAN2 induced a downregulation of the circular variant compared to controls (−1.24 ± 0.37 vs. 0.00 ± 0.29 log2 FC; Tukey’s adjusted *p* = 0.034; Supplementary Figure S3), while the linear variant was not affected (−0.42 ± 0.22 vs. 0.00 ± 0.42 log2 FC). The circ-C12orf29 siRNA downregulated circ-C12orf29 (−2.60 ± 0.30 vs. 0.00 ± 0.43 log2 FC; Tukey’s adjusted *p* = 0.004), without changing the expression of the linear variant (−0.31 ± 0.18 vs. −0.16 ± 0.70 log2 FC).

In pCFs, the transfection with the circ-RCAN2 siRNA selectively downregulated the circular (−2.18 ± 0.53 vs. −0.01 ± 0.47 log2 FC; Tukey’s adjusted *p* = 0.009; Supplementary Figure S3) but not the linear variant compared to controls (−0.10 ± 0.27 vs. 0.00 ± 0.13 log2 FC). Similarly, the siRNA-targeting circ-C12orf29 lead to a significant downregulation of circ-C12orf29 (−3.30 ± 0.98 vs. 0.00 ± 0.77 log2 FC; Tukey’s adjusted *p* = 0.006) compared to control cells. Although the linear transcript appeared to be minimally upregulated with respect to control cells, it was not upregulated when compared to cells transfected with the same dose of the SC siRNA (0.57 ± 0.10 vs. 0.48 ± 0.03 log2 FC; Tukey’s adjusted *p* = 0.429).

### 2.5. Effect of Circ-RCAN2 and Circ-C12orf29 on Cell Viability with and Without Hypoxia

**Figure 5:**
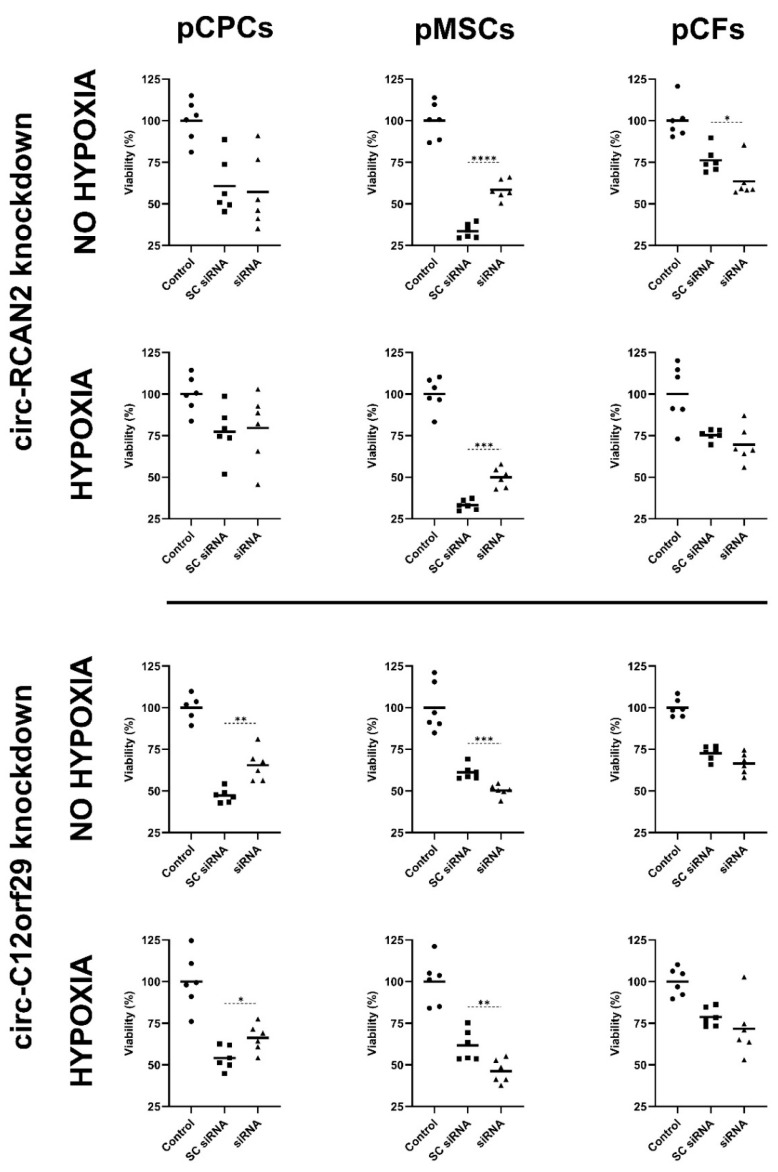
MTT assay for viability assessment after siRNA-mediated knockdown of circ-RCAN2 and circ-C12orf29. Change in viability of pCPCs, pCFs and pMSCs following a siRNA-mediated knockdown of circ-RCAN2 and circ-C12orf29 in either normal culture conditions or after 90 min of hypoxia. The level of significance is illustrated as * *p* = 0.01–0.05, ** *p* = 0.001–0.01, *** *p* = 0.0001–0.001 and **** *p* < 0.0001. Abbreviations: porcine cardiac progenitor cells (pCPCs), porcine mesenchymal stem cells (pMSCs), porcine cardiac fibroblasts (pCFs), small interfering RNA (siRNA).

siRNA-mediated knockdown of circ-RCAN2 had neither a significant effect on the viability of pCPCs cultured under normal conditions (SC siRNA: 60.68 ± 16.92% vs. siRNA: 57.12 ± 21.94%; unpaired *t*-test *p* = 0.759) nor after 90 min of hypoxia (SC siRNA: 77.30 ± 15.48% vs. siRNA: 79.62 ± 20.77%; unpaired *t*-test *p* = 0.831) compared to SC siRNA, as measured by the MTT assay (Figure 5). In contrast, a knockdown of circ-RCAN2 in pMSCs lead to a significant increase in viability in both the cells cultured under normal (SC siRNA: 33.58 ± 4.32% vs. siRNA: 58.52 ± 5.90%; unpaired *t*-test *p* < 0.001) and hypoxic conditions (SC siRNA: 32.25 ± 2.95% vs. siRNA: 49.95 ± 5.97%; unpaired *t*-test *p* < 0.001). In pCFs, the MTT assay indicated a significant decrease in cell viability after a knockdown of circ-RCAN2 under normoxic conditions (SC siRNA: 76.22 ± 7.48% vs. siRNA: 63.55 ± 10.87%; Mann–Whitney U test *p* = 0.041). Although a numerically similar effect was present under 90 min of hypoxia, it was not statistically significant (SC siRNA: 75.38 ± 3.30% vs. siRNA: 69.55 ± 10.91%; unpaired *t*-test with Welch’s correction *p* = 0.258).

A knockdown of circ-C12orf29 increased pCPC viability both under normal conditions (SC siRNA: 47.20 ± 4.22% vs. siRNA: 65.45 ± 9.51%; unpaired *t*-test *p* = 0.002), and after 90 min of hypoxia (SC siRNA: 54.10 ± 7.00% vs. siRNA: 66.25 ± 8.25%; unpaired *t*-test *p* = 0.020) compared to SC siRNA (Figure 5). Contrastingly, in MSCs, a knockdown of circ-C12orf29, led to a significantly decreased viability in both the cells cultured under normal conditions (SC siRNA: 61.13 ± 4.35% vs. siRNA: 50.30 ± 3.54%; unpaired *t*-test *p* < 0.001) and cells after 90 min of hypoxia (SC siRNA: 61.58 ± 9.32% vs. siRNA: 46.15 ± 7.06%; unpaired *t*-test *p* = 0.009). A decreased, albeit nonsignificant, viability was similarly seen in pCFs after circ-C12orf29 knockdown both under normal conditions (SC siRNA: 72.62 ± 4.15% vs. siRNA: 66.53 ± 6.20%; unpaired *t*-test *p* = 0.074) and after 90 min of hypoxia (SC siRNA: 78.70 ± 5.60% vs. siRNA: 71.67 ± 16.90%; unpaired *t*-test with Welch’s correction *p* = 0.370).

### 2.6. Scratch Wound Healing Assay

**Figure 6:**
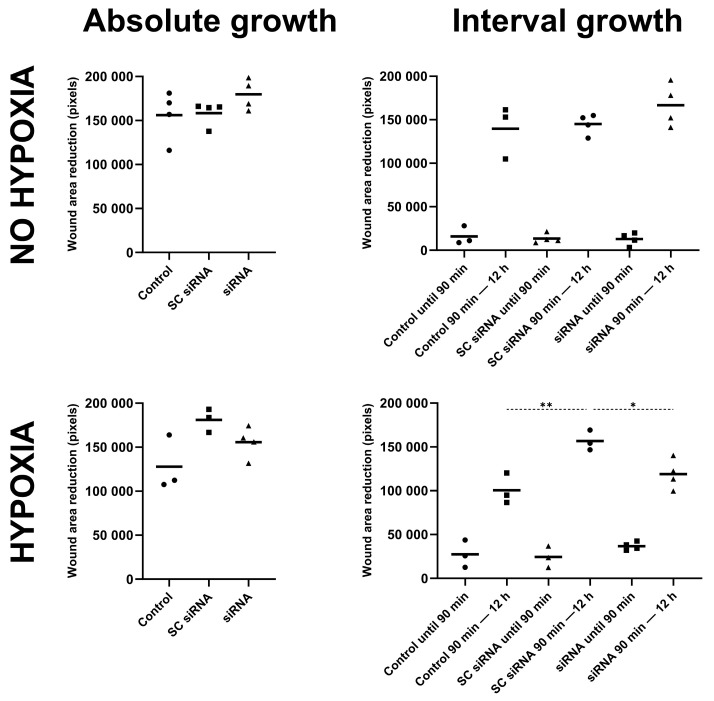
Scratch wound healing assay following transfection with siRNA-targeting circ-C12orf29 in pCPCs. Wound healing assay in pCPCs following transfection with an SC siRNA or a siRNA-targeting circ-C12orf29 and controls either cultured in normal conditions or with 90 min of hypoxia. Left panels: Wound area closure from scratch creation until 12 h. Right panels: Wound area closure from scratch creation until 90 min and from 90 min until 12 h. The level of significance is illustrated as * *p* = 0.01–0.05, ** *p* = 0.001–0.01. Abbreviations: porcine cardiac progenitor cells (pCPCs), porcine mesenchymal stem cells (pMSCs), porcine cardiac fibroblasts (pCFs), small interfering RNA (siRNA).

A knockdown of circ-C12orf29 had no effect on the absolute wound area closure compared to non-transfected controls or cells transfected with SC siRNA as assessed by the scratch assay (Figure 6). However, when observing the more granular data in cells where the scratch was created prior to 90 min of hypoxic treatment, the SC siRNA-transfected cells demonstrated a significantly higher rate of wound closure between the termination of hypoxia and 12 h compared to control cells (156,784 ± 11,432 vs. 100,603 ± 17,509 pixels for the SC siRNA versus controls; Sidak’s test *p* = 0.001), whereas the cells transfected with the siRNA-targeting circ-C12orf29 demonstrated a significantly reduced area of wound closure compared to their SC siRNA counterparts (156,784 ± 11,432 vs. 118,912 ± 17,013 pixels for the SC siRNA versus circ-C12orf29 siRNA; Sidak’s test *p* = 0.016).

### 2.7. Effect of Circ-RCAN2 and Circ-C12orf29 Knockdown on the Expression of Predicted miRNA Targets

**Figure 7:**
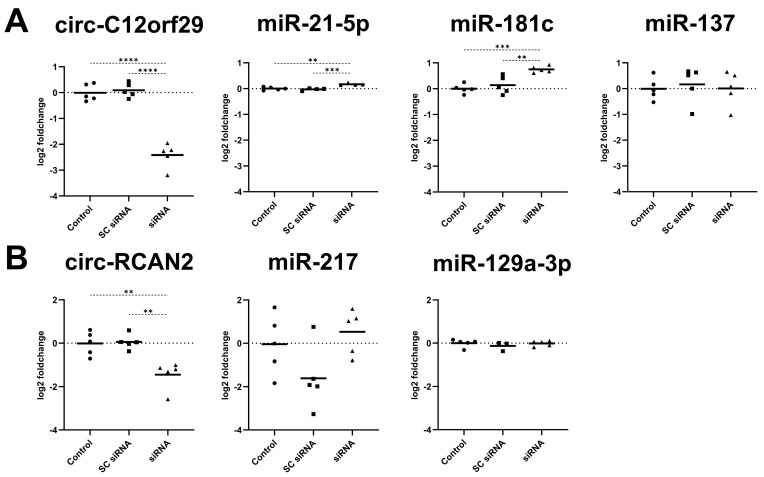
Expression of predicted miRNA targets of circ-C12orf29 and circ-RCAN2 following siRNA-mediated knockdown. (**A**) Change in expression of circ-C12orf29 and predicted targets ssc-miR-21-5p, ssc-miR-181c and ssc-miR-137 in pCPCs after siRNA-mediated knockdown of circ-C12orf29 compared with SC siRNA and controls. (**B**) Change in expression of circ-RCAN2 and predicted targets ssc-miR-217 and ssc-miR-129a-3p in pCPCs after siRNA-mediated knockdown of circ-RCAN2 compared with SC siRNA and controls. The level of significance is illustrated as ** *p* = 0.001–0.01, *** *p* = 0.0001–0.001 and **** *p* < 0.0001. Abbreviations: porcine cardiac progenitor cells (pCPCs), small interfering RNA (siRNA).

The circ-C12orf29 knockdown was again confirmed, as the transfection of pCPCs with the siRNA led to a significant downregulation of circ-C12orf29 (−2.42 ± 0.47 vs. 0.00 ± 0.33 log2 FC; Tukey’s adjusted *p* < 0.001) compared to controls (Figure 7A). In parallel, this knockdown of circ-C12orf29 led to a significant upregulation of predicted targets ssc-miR-21-5p (0.17 ± 0.04 vs. 0.00 ± 0.06 log2 FC; Tukey’s adjusted *p* = 0.002) and ssc-miR-181c (0.75 ± 0.13 vs. 0.00 ± 0.18 log2 FC; Tukey’s adjusted *p* < 0.001) compared to controls. The knockdown had no effect on the expression of ssc-miR-137 (0.01 ± 0.67 vs. −0.01 ± 0.43 log2 FC; Tukey’s adjusted *p* = 0.999).

The efficient knockdown of circ-RCAN2 by the siRNA was confirmed using qPCR, which showed a significant downregulation of circ-RCAN2 (−0.01 ± 0.55 vs. −1.45 ± 0.64 log2 FC; Tukey’s adjusted *p* = 0.003) compared to controls (Figure 7B). The knockdown of circ-RCAN2 had no effect on the expression of the predicted targets ssc-miR-217 or miR-129a-3p.

## 3. Discussion

circRNAs are a novel addition to the complex mechanisms surrounding the pathophysiology of myocardial infarction, post-infarction remodeling and regeneration [6]. In this study, we investigated CDR1as, circ-RCAN2 and circ-C12orf29, three circRNAs whose differing expression patterns were described in the context of a large animal model of reperfused myocardial infarction by our group [14,15]. Despite the initial pathophysiological insights gained on the nature and regulation of these circRNAs, including their successful amplification from cardiac tissue, concrete insights on their precise regulation following varying intervals of hypoxia and their role as downstream regulators had not been performed. Our results now confirm not only the circular nature of these candidate circRNAs but also highlight cell-specific expression patterns in response to nutrient deprivation and hypoxia, distinct effects on cell viability and downstream miRNA targets.

Amplification of the predicted BSJ has previously been confirmed by our group via Sanger sequencing of qPCR products from myocardial tissue [11,12]. However, the circular nature of the transcripts, i.e., their resistance to exonuclease treatment, and their cell-specific expression patterns had not been confirmed. In this study, we demonstrate that all three candidates are amplified by qPCR following prolonged RNase R depletion, thus proving their circular nature in line with common principles for validation of novel circRNA candidates [24]. Sanger sequencing of untreated and RNase R depleted product both confirmed the amplification of an identical BSJ for all three circ-RNAs. On agarose gel electrophoresis, band intensity was visually comparable for circ-RCAN2 and circ-C12orf29, regardless of RNase R treatment, implying RNase R resistance. In contrast, CDR1as band intensity decreased with treatment, indicating a certain degree of sensitivity to RNase R treatment at our chosen concentration, which may be explained by prolonged incubation times, which may lead to a degree of degradation of some circRNAs [24].

To investigate the expression of our chosen circRNAs at specific intervals of hypoxia and nutrient deprivation mimicking their regulation in myocardial infarction, we selected an in vitro approach investigating cells with potential roles in post-infarction regenerative processes. CPCs are a diverse population of resident stem-like cells of the developing and adult heart which have been at the forefront of a number of studies investigating their potential supportive contribution to regenerative processes following myocardial infarction, which in recent years has particularly focused on paracrine mechanisms [25,26]. Similarly, MSCs, and among them bone-marrow-derived MSCs, have been widely studied for their regenerative potential following myocardial infarction, also significantly conveyed via the paracrine route [27,28]. CFs, which constitute a significant portion of the resident cardiac cell population, are crucial in the normal functioning of the heart, the healing response, but also the adverse remodeling following myocardial infarction [29]. In our study, subjecting pCPCs, pMSCs and pCFs to serum deprivation and varying intervals of hypoxia to mimic acute and prolonged ischemic conditions resulted in cell-specific expression patterns of CDR1as, circ-RCAN2 and circ-C12orf29. Specifically, in pCPCs, serum deprivation on its own induced an upregulation of all investigated circRNAs, with an additional increase in CDR1as after 1 h of hypoxia, which was not seen in the other two candidates. Importantly, all candidate circRNAs demonstrated an additional spike in expression following a prolonged hypoxic interval of 48 h. A similar pattern could be observed in MSCs, where circ-RCAN2 and circ-C12orf29 were significantly upregulated with 48 h of hypoxia, potentially pointing towards the involvement of these molecules past the immediate short-term response to nutrient deprivation and hypoxia. Nevertheless, despite an initial increase with serum deprivation, all circRNAs were downregulated in our studied MSC population in the short-term hypoxia phase, which may potentially stem from the different durations of serum deprivation prior to hypoxia. In our population of pCFs, the differential regulation of all studied circRNAs was less pronounced compared to pCPCs and pMSCs, with no clear unifying trends in their expression with prolonged hypoxia, yet the omitted serum deprivation of pCFs in our setting must be considered. Overall, these findings align with previous observations by our group, wherein circ-RCAN2 was upregulated in pCPCs subjected to intervals of hypoxia spanning up to 8 h [15]. However, they contrast the in vivo findings, where at three days post-closed-chest reperfused porcine infarction, circ-C12orf29 and circ-RCAN2 were downregulated in the infarcted area [15]. These are nonetheless fundamentally different settings, with bulk myocardium consisting of multiple cell types sampled on the third day following reperfused myocardial infarction on the one hand and a cell-specific approach with hypoxia intervals up to 48 h on the other.

Having confirmed the differential regulation of CDR1as, circ-RCAN2 and circ-C12orf29, we now focused on the effects of the two novel candidates—circ-RCAN2 and circ-C12orf29—on cell viability under normoxic conditions and following 90 min of hypoxia, mimicking a commonly observed pre-reperfusion interval in the clinical setting [2]. Notably, we observed that the effect of the knockdown itself on the viability of the cells superseded the effect of the comparatively short 90 min interval of hypoxia, i.e., that the direction of the effect remained the same regardless of the presence of hypoxic or normoxic conditions. Importantly, the effects on viability did not appear to correlate with the degree of dysregulation under serum deprivation or hypoxia. circ-RCAN2 appears to have protective properties in pCFs, as its knockdown reduced cell viability, while simultaneously inducing potentially detrimental effects on pMSC survival. In contrast, circ-C12orf29 appears to benefit both pMSC survival and potentially pCF survival, while its knockdown appeared to increase viability of pCPCs. The discrepancy in these findings could be explained by multiple factors, ranging from the capture of a “reperfusion” phase by the MTT assay, which likely differs by the selected cell type, as well as a different interaction with downstream targets in each of the cell types. In contrast, the scratch assay did not demonstrate a significant effect on scratch healing in pCPCs treated with a siRNA-targeting circ-C12orf29 in a 12 h timeframe. However, when stratifying cell growth before and after hypoxia, the post-hypoxia interval was characterized by a poorer growth of the siRNA-transfected cells compared to SC siRNA controls, which could be attributed to either a lingering hypoxic influence or the “reperfusion” conveyed by the introduction of new medium. Thus, our findings highlight that, in addition to their cell-specific regulation, the effects of our novel circRNA candidates on cell survival also appear to be cell-type-specific.

While the first publication by our laboratory postulated miR-7 as the target of CDR1as, as was already established in other organisms, and delivered some initial evidence for the negative correlation in their expression, mechanistic studies on circ-RCAN2 and circ-C12orf29 had not yet been conducted [9,14]. Our findings now indicate that a siRNA-mediated knockdown of circ-C12orf29 in pCPCs leads to a significant upregulation of ssc-miR-21-5p and ssc-miR-181c, identifying these two miRNAs as potential targets of circ-C12orf29. Notably, the upregulation of ssc-miR-181c was numerically substantially higher than that of ssc-miR-21-5p, although this should be observed in the context of the high comparative abundance of ssc-miR-21-5p. In the available literature, miR-181c-5p was found to mediate proinflammatory effects of hypoxia reperfusion injury in rat cardiomyoblast cells, and its inhibition led to an attenuation of the phenotype of heart failure in a subsequent murine model, albeit at the cost of renal impairment induced by the applied antagonist [30,31]. Similarly, the inhibition of miR-181c-5p has also been demonstrated to attenuate hypoxia-reperfusion-mediated apoptosis of rat cardiomyoblasts [32]. Another study indicated that miR-181c targets mitochondrial cytochrome c oxidase subunit 1 in mitochondria and increases reactive oxygen species production as a potential downstream pathway [33]. In contrast, miR-21-5p has, despite some degree of conflicting information, largely been attributed cardioprotective and antiapoptotic properties in preclinical studies, also in the setting of ischemia/hypoxia [34,35,36,37,38,39]. The antiapoptotic effects of miR-21-5p in the context of exosomes derived from mesenchymal stem cells have been linked with the protein PTEN in both cancer and non-cancer settings [40,41]. In cardiac fibroblasts, the ERK-MAP kinase has been implicated in miR-21 signaling, leading to increased fibroblast survival [42]. In cardiac progenitor cells, hypertrophy has been associated with an increased expression of miR-21 [43]. On the other hand, miR-21 produced in exosomes of cardiac progenitor cells attenuates cardiomyocytes death via targeting PDCD4 [44]. Notably, we were not able to confirm any targets for circ-RCAN2. Neither miR-217, which was a predicted target according to our algorithm, nor miR-129a-3p, identified as a target by previous unpublished research by our group, showed any deviation after successful circ-RCAN2 knockdown in our chosen setting. However, it is important to note that the circRNA–miRNA regulation could be specific to each cell type, and the role of each miRNA might change according to the specific setting.

Despite the novel insights gained by our study, some limitations need to be considered when interpreting our results. First, as this is a pilot study on the selected novel circRNA candidates, further studies are needed to validate our findings, which might involve Northern blots of these circRNA candidates not conducted as part of this study. Second, our fibroblasts did not tolerate serum deprivation with prolonged hypoxia, thus requiring careful interpretation of findings across cell types. Third, although the MTT assay is widely used as a measure of cell viability, it is rather a reflection of a sum of factors, including metabolism and MTT-toxicity [45]. Fourth, the interpretation of our scratch assay is somewhat limited by the use of a comparatively high serum concentration necessary for the survival of the chosen cell type, which in turn means we likely measured a combination of migration and proliferation. Fifth, we merely assessed potential miRNA targets for two of the circRNAs, although this is only one of the many mechanisms by which circRNAs exert their downstream effects. circRNAs can also exert a multitude of additional effects including protein sponging or stabilization, they can facilitate or regulate protein–protein contacts or even be translated themselves [46]. Having established the relevance of CDR1as, circ-RCAN2 and circ-C12orf29 in hypoxia, these are all viable avenues for further research on downstream targets. Finally, the clinical relevance of these findings hinges on their further translation, i.e., the identification and confirmation of human orthologs and their downstream targets, which may lead to the identification of novel pathways involved in post-infarction remodeling, as well as potential treatment targets.

In conclusion, our study confirms the circular nature of CDR1as, circ-RCAN2 and circ-C12orf29. Based on our findings, these candidate circRNAs appear to be dysregulated in a time-dependent and cell-type-specific manner following hypoxia and nutrient deprivation, exhibit cell-type-specific effects on viability under normoxic and hypoxic conditions and, in the case of circ-C12orf29, appear to target miR-21-5p and miR-181c, making them promising candidates for future research in the field of myocardial infarction.

## 4. Materials and Methods

### 4.1. Cell Culture and Cell Characterization

Analyses within this study focused on pCPCs, pCFs and pMSCs. Unless otherwise stated, cells were cultured at 37 °C, 5% CO_2_ and moderate humidity. The source or isolation protocols, as well as the culturing conditions for the cells used in the experiments, are set out in the Supplementary Methods [43,47]. Cells were characterized by immunofluorescence according to a previously published protocol [15], described in the Supplementary Methods.

### 4.2. RNA Isolation and cDNA Synthesis

Total RNA was isolated from cells using the RNeasy Mini Kit (QIAGEN GmbH, Hilden, Germany) according to the manufacturer protocol. Subsequently, cDNA synthesis was performed using the QuantiTect Reverse Transcription Kit (QIAGEN GmbH, Hilden, Germany) according to the manufacturer protocol.

### 4.3. qPCR

qPCR was performed using the QuantiTect SYBR Green PCR kit (Qiagen, Hilden, Germany). The divergent primers used for the detection of the BSJ of CDR1as (circAtlas ID sus-CDR1_0001, conserved from human CDR1as, referred to henceforth as CDR1as), circ-RCAN2 (circAtlas ID sus-RCAN2_0004, conserved from human hsa-RCAN2_0001, referred to henceforth as circ-RCAN2) and circ-C12orf29 (circAtlas ID sus-C12orf29_000, conserved from human hsa-C12orf29_0003, referred henceforth to as circ-C12orf29), as well as the primers used for the detection of HPRT (used as reference) were previously published by our group [14,15]. The primers used for the amplification of HIF-1α were previously described: forward primer 5′-ACCTGAGCCTAACAGTCCCAGTG-3′, reverse primer 5′-TTCTTTGCCTCTGTGTCTTCAGCAA-3′ [48].

### 4.4. RNase R Treatment

To confirm that the chosen targets are indeed circular RNA molecules, we subjected the total isolated RNA to RNase R treatment with 10 U of RNase R (Lucigen, UK) per 800 ng RNA. The incubation step was performed at 43 °C for 2 h to ensure complete degradation of the selected linear control. The resulting RNA was used for cDNA synthesis and qPCR, as described above.

### 4.5. Agarose Gel Electrophoresis

Gel electrophoresis of PCR products generated from undigested and RNase R digested RNA was performed in a 2% agarose gel according to a standardized protocol as described within the Supplementary Methods. Raw data are shown in Supplementary Figure S4.

### 4.6. DNA Precipitation and Sanger Sequencing

qPCR products were precipitated according to an adjusted ethanol-based protocol with 0.3 M sodium acetate [49]. Sanger sequencing was performed by Microsynth Austria GmbH (Vienna, Austria). Sanger sequencing traces were visualized using the FinchTV software (1.4.0, Geospiza, Inc., Seattle, WA, USA).

### 4.7. In Vitro Hypoxia Model

To investigate the differential regulation of CDR1as, circ-RCAN2 and circ-C12orf29 under ischemic conditions in vitro, cells were first cultured in the full appropriate medium until confluency. Prior to hypoxia, medium was replenished with the appropriate serum-free medium in pCPCs (approximately six hours prior to hypoxia) and pMSCs (approximately three hours prior to hypoxia), but not in pCFs (due to massive cell death with prolonged serum deprivation in combination with hypoxia at later timepoints). The plate and a water dish were placed into the hypoxia chamber (STEMCELL Technologies Germany GmbH, Cologne, Germany) which was subsequently flushed with a <1% oxygen combination from a nitrogen and oxygen tank for at least two minutes to ascertain complete filling with the appropriate gas concentration. The chamber valves were sealed and the chamber placed into the incubator at 37 °C for 1, 2 and 3 h to mimic acute ischemia and 12 and 48 h to mimic prolonged ischemia. Cells from the same line cultured under normal oxygen and serum conditions served as controls. After the appropriate interval, the medium was immediately removed, cells were washed with PBS, and RNA was isolated without delay. The workflow of the assay is illustrated in Supplementary Figure S1.

### 4.8. Small Interfering RNA Design

To confirm the effect of circ-RCAN2 and circ-C12orf29 on the proposed miRNA targets, we designed and tested small interfering RNAs (siRNA) spanning the BSJ of the respective circRNA to ensure specific knockdown of the circular transcript. siRNA-mediated knockdowns were performed using forward lipid-mediated transfection with Lipofectamine^®^ RNAiMAX (Thermo Fisher Scientific, Vienna, Austria) in line with the manufacturer’s protocol. Following siRNA-mediated circRNA knockdown, total RNA was isolated from the cells 24 h after siRNA application. The selected siRNA sequences, design rationale and applied dose of siRNA and transfection reagent are listed in the Supplementary Methods [50,51,52,53]. siRNA experiments to induce CDR1as knockdown were not performed as data regarding downstream miRNA targets of CDR1as is already available [9].

### 4.9. MTT and Scratch Wound Healing Assays

A standard MTT (3-(4,5-Dimethylthiazol-2-yl)-2,5-Diphenyltetrazolium Bromide) assay was applied to test the effect of circ-RCAN2 and circ-C12orf29 on cell viability under normal oxygen conditions and hypoxia, as shown in the Supplementary Methods and Supplementary Figure S2 [54]. The viability of the cells transfected with the appropriate siRNA and SC siRNA was calculated with respect to non-transfected controls while subtracting the dimethyl sulfoxide background absorbance.

A scratch wound healing assay conducted on pCPCs following a siRNA-mediated knockdown of circ-C12orf29 as described in the Supplementary Methods. The borders of the cell layer were manually traced in the cellSens imaging software (v.1.17, Olympus, Tokyo, Japan). To standardize the observed scratch area, an area at a specified distance from the reference line was considered. The area of the scratch in this field of view was recorded in pixel units. A mean from two measurements performed by the same operator at different times was used for calculations with each image.

### 4.10. Identification of miRNA Targets

The miRanda v3.3a microRNA Target Scanning Algorithm was used to predict potential miRNA targets of circ-RCAN2 and circ-C12orf29 with an open gap penalty of −9.0, a gap extend penalty of −4.0, a score threshold of 140, an energy threshold of −18 kcal/mol and a scaling parameter of 4.0, as well as the requirement for a strict alignment in the seed region [55]. Predicted miRNAs were manually screened for human orthologs using the Ensembl database [56]. Only miRNAs with human orthologs were further considered for verification as the targets of the respective circRNA.

### 4.11. Confirmation of miRNA Targets

pCPCs used for the confirmation of potential miRNA targets were cultured and a knockdown of circ-RCAN2 and circ-C12orf29 was induced as described above. A total of 24 h after the knockdown, total RNA including miRNA and other small RNAs was manually isolated using the miRNeasy mini kit (Qiagen, Hilden, Germany), according to the manufacturer protocol. cDNA synthesis was conducted either using the QuantiTect Reverse Transcription kit (Qiagen, Hilden, Germany) for the verification of the circRNA knockdown or the miRCURY LNA RT kit (Qiagen, Hilden, Germany) for the analysis of the miRNA levels from the RNA of the same samples. qPCR was performed either using the QuantiTect SYBR Green PCR kit (Qiagen, Hilden, Germany) to verify the knockdown of the target circRNA molecules with the primers described above or the miRCURY LNA SYBR Green PCR kit (Qiagen, Hilden, Germany). The qPCR for the verification of potential target miRNAs of circ-RCAN2 (ssc-miR-217) or circ-C12orf29 (ssc-miR-21-5p, ssc-miR-181c, ssc-miR-137) was performed with ssc-let-7a as the reference gene to account for differences arising between mRNA and small microRNA during isolation, reverse transcription and qPCR. Primers were purchased from the manufacturer (Qiagen, Hilden, Germany).

### 4.12. Statistical Analysis

All statistical analyses were performed by GraphPad Prism Software Version 10.1.2 (La Jolla, CA, USA). Continuous variables were reported as mean and standard deviation (SD) and compared by the Student’s t-test or the Mann–Whitney U test, depending on the presence of a normal distribution, as assessed using the Shapiro–Wilk test and visual inspection of density plots. For variables with a low number of replicates (n < 5) where normal distribution could be assumed due to the biological properties of the tested variable, variables were considered normally distributed. Outliers were removed in case they were either deemed biologically implausible, the consequence of technical error or identified by Grubb’s test. Multiple group comparisons were conducted using ANOVA and Tukey’s multiple comparisons test or Welch’s ANOVA (when differing variances were identified by the Brown–Forsythe test) followed by Dunett’s T3 correction. A two-sided *p*-value of 0.05 was considered statistically significant.

### 4.13. Ethical Considerations

Due to the strictly in vitro study design, no ethical approval was required for the conduction of this study.

## Data Availability

Data is available with publication upon reasonable request from the corresponding author.

## Supplementary Materials

The following supporting information can be downloaded at: https://www.mdpi.com/article/10.3390/ijms262110334/s1.

## Abbreviations

circRNA, circular RNA; miRNA, microRNA; pCPCs, porcine cardiac progenitor cells; pMSCs, porcine mesenchymal stem cells; pCFs, porcine cardiac fibroblasts; Nkx2.5, NK2 homeobox 5; αSMA, alpha smooth muscle actin; Isl-1, Islet-1; FSP1, S100A4/Fibroblast-specific protein 1; BSJ, backsplice junction; siRNA, small interfering RNAs; SC siRNA, scrambled siRNA; MTT, 3-(4,5-Dimethylthiazol-2-yl)-2,5-Diphenyltetrazolium Bromide; bp, base pairs; HIF1α, hypoxia inducible factor 1 alpha.
