# Supplementary material for: Cardiac Circular RNAs CDR1as, Circ-RCAN2, Circ-C12orf29 Show Cell-Specific Hypoxia-Induced Dysregulation and Distinct In Vitro Effects
Source: Int J Mol Sci. 2025 Oct 23;26(21):10334. doi: 10.3390/ijms262110334 (PMC12607399; doi:10.3390/ijms262110334)
Supplement: Supplementary file 1 [file ijms-26-10334-s001.zip › ijms-3919276-supplementary.pdf]

SUPPLEMENTAL MATERIALS

Cardiac circular RNAs CDR1as, circ-RCAN2, circ-C12orf29 show cell-specific hypoxia-induced dysregulation and distinct *in vitro* effects

Ena Hasimbegovic, MD[a] (ena.hasimbegovic@meduniwien.ac.at)
Dominika Lukovic, PhD[a] (dominika.lukovic@meduniwien.ac.at)
Nina Kastner, MSc[a,b] (nina.kastner@meduniwien.ac.at)
Benedikt S Hofer, MD[c] (benedikt.s.hofer@meduniwien.ac.at)
Andreas Spannbauer, MD[a] (andreas.spannbauer@meduniwien.ac.at)
Denise Traxler, MD[a,d] (denise.traxler-weidenauer@meduniwien.ac.at)
Julia Mester-Tonczar, PhD[a] (julia.mester-tonczar@meduniwien.ac.at)
Kevin Hamzaraj, MD[a] (kevin.hamzaraj@meduniwien.ac.at)
Emilie Han, MD[a,e] (emilie.han@meduniwien.ac.at)
Martin Riesenhuber, MD, PhD[a] (martin.riesenhuber@meduniwien.ac.at)
Babette Maleiner, MSc[a] (babette.maleiner@meduniwien.ac.at)
Katrin Müller-Zlabinger, PhD[a,#] (katrin.mueller-zlabinger@meduniwien.ac.at)
Mariann Gyöngyösi, MD, PhD[a,#,*] (mariann.gyongyosi@meduniwien.ac.at)

[a] Division of Cardiology, Department of Internal Medicine II, Medical University of Vienna, Vienna 1090, Austria
[b] Institute of Pharmacology, Center of Physiology and Pharmacology, Medical University of Vienna, Vienna 1090, Austria
[c] Division of Gastroenterology and Hepatology, Department of Internal Medicine III, Medical University of Vienna, Vienna 1090, Austria
[d] Department of Oral and Maxillofacial Surgery, Medical University of Vienna, Vienna 1090, Austria
[e] Department of Transfusion Medicine and Cell Therapy, Medical University of Vienna, Vienna 1090, Austria
[#] Contributed equally

***Corresponding Author:**
Univ.Prof. Dr. Mariann Gyöngyösi
Medical University of Vienna
Department of Internal Medicine II
Division of Cardiology
Waehringer Guertel 18-20
A-1090 Vienna, Austria
Tel.: +43-(0)1-40400-46140
mariann.gyongyosi@meduniwien.ac.at

TABLE OF CONTENTS

SUPPLEMENTAL METHODS....................3

Cell culture....................3

Cell characterization....................4

Agarose gel electrophoresis....................6

Small interfering RNA design....................7

MTT assay....................8

Scratch wound healing assay....................9

SUPPLEMENTAL FIGURES....................10

Supplemental Figure S1. Flowchart depicting the workflow of the hypoxia assay....................10

Supplemental Figure S2. Flowchart depicting the workflow of the MTT assay....................10

Supplemental Figure S3. siRNA-mediated knockdown of circ-RCAN2 and circ-C12orf29. 11

Supplemental Figure S4. Agarose gels used for Figure 2 in the main manuscript....................12

SUPPLEMENTAL TABLES....................13

Supplemental Table S1. HIF1α, CDR1as, circ-RCAN2 and circ-C12orf29 expression after progressively longer intervals of hypoxia....................13

SUPPLEMENTAL REFERENCES....................14

SUPPLEMENTAL METHODS

Cell culture

pCPCs used in our experiments were previously isolated by our laboratory according to the protocols described by Zlabinger et al.[1] pCPC medium was made up of an equal parts mixture of Dulbecco's Modified Eagle Medium (DMEM-HG) 4.5 g/L glucose (Sigma-Aldrich, USA) and Medium 199 (Sigma-Aldrich, Germany), supplemented with 10% FBS Superior (Sigma-Aldrich, Germany) and 1% Penicillin/Streptomycin (P4458, Sigma-Aldrich, Germany). pMSCs were previously isolated by our laboratory according to the following procedure adapted from previously published protocols[2]: 1 mL of a 2000 U/mL heparin solution (Sigma-Aldrich, Germany) was added per 20 mL of porcine bone marrow aspirate collected from domestic pigs following flushing of instruments with the heparin solution. The heparin-bone marrow aspirate solution was carefully added to 50 mL centrifuge tubes containing 15 mL of Ficoll-Paque™ PREMIUM (Cytiva, USA). The tubes were centrifuged at 445 *g* and room temperature for 10 minutes. The bone marrow fraction was carefully collected into a new tube and washed with PBS twice, followed each time by centrifugation at 300 *g* and room temperature for 5 minutes. pMSCs were cultured in medium consisting of DMEM-HG 4.5 g/l glucose (Sigma-Aldrich, USA) supplemented with 10% FBS Superior (Sigma-Aldrich, Germany) and 1% Penicillin/Streptomycin (P4458, Sigma-Aldrich, Germany). Cryopreserved primary pCFs (P-6049, Cell Biologics, Illinois, USA) were purchased from the manufacturer and cultured in medium consisting of DMEM-HG 4.5 g/L glucose (sigma-Aldrich, USA) supplemented with 10% FBS Superior (Sigma-Aldrich, Germany) and 1% Penicillin/Streptomycin (P4458, Sigma-Aldrich, Germany).

Cell characterization

Cells were seeded on an 8-well chamber slide (ibidi GmbH, Germany) in full medium until adherent. Medium was removed and cells were washed three times with PBS. PBS was removed and cells were fixated with a 4% paraformaldehyde solution (SAV Liquid Production GmbH, Flintsbach a. Inn, Germany) without methanol in PBS for 10 minutes at 37°C and washed three times with PBS. Where required, permeabilization was performed by incubating the cells with 0.1% Triton X-100 (Sigma Aldrich, St. Louis, MO, United States) in PBS for 10 minutes at room temperature. Blocking was performed with 2% Bovine Serum Albumin (BSA) (Sigma Aldrich, St. Louis, MO, United States) in PBS for 1 hour at room temperature. Blocking buffer was removed and the primary antibody diluted in the appropriate concentration in 0.1% BSA in PBS was added and incubated overnight at 4°C. The primary antibodies and dilutions were as follows: pCFs: Vimentin Antibody ab128507 (abcam, Cambridge, United Kingdom) 1:100, S100A4/Fibroblast-specific protein 1 antibody LS-B2381 (LifeSpan Biosciences, United States) 1:100, CD90/Thy 1 antibody 328102 (BioLegend, United States) 1:200; pCPCs: NK2 homeobox 5 antibody ab106923 (abcam, Cambridge, United Kingdom) 1:200, Alpha smooth muscle Actin Antibody ab5694 (abcam, Cambridge, United Kingdom) 1:300, Islet-1 antibody orb251477 (biorbyt, Cambridge, United Kingdom) 1:100, c-kit antibody 313202 (Biolegend, United States) 1:100; pMSCs: CD90/Thy 1 antibody 328102 (BioLegend, United States) 1:200, direct labeling with CD29 antibody (Exbio, Prague, Czech Republic) 1:100, direct labeling with CD44 antibody (Exbio, Prague, Czech Republic) 1:100. Cells were washed three times with PBS and incubated with the appropriate secondary antibody (Goat anti-Mouse Antibody A-11001 [Thermo Fisher Scientific, United States] 1:1000, Rabbit anti-Goat Antibody A-11078 [Thermo Fisher Scientific, United States] 1:500, Goat anti-Rabbit Antibody A-11008 [Thermo Fisher Scientific, United States] 1:500) diluted in 0.1% BSA in PBS with 1:40 phalloidin (Thermo Fisher Scientific, United States) for 45 minutes at room temperature

in the dark. Cells were washed with PBS and incubated with a 1:2500 dilution of Hoechst 33342 for 5 minutes in the dark. Cells were washed with PBS. Glass coverslips were mounted with Fluoroshield (Abcam, United Kingdom) and imaged immediately on the FLoid™ Cell Imaging Station (Thermo Fisher Scientific, United States).

Agarose gel electrophoresis

A 2% agarose solution from 2 g of agarose (A4718-100G, Sigma-Aldrich Handels Gmbh, Vienna, Austria) in running buffer (TAE or TBE) (bioWORLD, Ohio, United States) was heated for five minutes in the microwave while periodically vigorously swirling the flask to allow all bubbles to move to the surface. After cooling to 60°C, 5 µL of GelRed® (Hayward, USA) was added to the gel and the gel was cast in a 7x7 cm tray. A comb with 3mm width was used. The gel was cooled at room temperature for thirty minutes, then submerged in buffer (TAE or TBE). PCR solutions were combined with RNase free water and 10x FastDigest Green Buffer (Thermo Fisher Scientific, United States) before being added to individual wells. QuickLoad 100 bp DNA Ladder (New England Biolabs GmbH, Frankfurt am Main, Germany) was added to one well as reference. The gel was run at 70V for 1 hour in the Mini-Sub Cell GT Cell (Bio-Rad Laboratories Ges.m.b.H., Vienna, Austria). The gel was imaged using Azure c600 (Azure Biosystems, Dublin, CA, USA).

Small interfering RNA design

We opted for 21 nucleotide siRNA with a 19 nucleotide duplex and a deoxythymidine dinucleotide (dTdT) 3’ overhang on the sense and antisense strand.[3] The siRNA used to downregulate circ-RCAN2 (sense strand [ss] 5'-CAA AUU UUU CCU GGU CCU A dTdT-3', antisense strand [as] 3'-dTdT GUU UAA AA A GGA CCA GGA U-5') was previously designed by our laboratory (data unpublished). The design of the siRNA for the knockdown of circ-C12orf29 (ss 5'-CUG ACC AUA UGG GUU UCC A dTdT-3', as 3'-dTdT GAC UGG UAU ACC CAA AGG U-5') was in line with the rules set out by Ui-Tei et al.[4] Scrambled siRNAs (SC siRNA) for circ-RCAN2 (ss 5'-AUU CUG CUU ACG CUA CUC U dTdT-3', as 3'-dTdT UAA GAC GAA UGC GAU GAG A-5') and circ-C12orf29 (ss 5'-GCU CUU GCG UAU AAC AGU C dTdT-3', as 3'-dTdT CGA GAA CGC AUA UUG UCA G-5') were designed using Genscript. All siRNA were screened using the Basic Local Alignment Search Tool.[5] All siRNA were synthesized by Microsynth AG (Balgach, Switzerland). Dose finding experiments were performed to identify the optimal concentration of the chosen siRNA, confirm that the SC siRNA does not influence the target circRNA, and that the siRNA applied does not regulate the linear transcript. The final dose for siRNA used in pCPCs and pMSCs was 28 µmol of siRNA and 2µl Lipofectamine® RNAiMAX per 100 µl Opti-MEM®. In pCFs 16 µmol siRNA and 2µl Lipofectamine® RNAiMAX per 100 µl Opti-MEM® was used.

Primers designed using the NCBI’s Blast to amplify the linear transcript but not the circular counterpart were used to quantify the expression of linear RCAN2 (fw primer 5'-AAG CCT CTC GGA CAA AAG GG-3', rv primer 5'-TTG TCA CCG ATG TCT CTG GC-3') and C12orf29 (fw primer 5'-AGA GGG AGC ATC AGC CAT TTA-3', rv primer 5'-CTG TCT AGC CGA GCC CAA AG-3'). The primers were synthesized by Microsynth Austria GmbH (Vienna).[6]

MTT assay

Cells were plated in 48-well plates containing the appropriate medium one day prior to transfection. Wells not containing cells were filled with water to prevent dehydration. 48-well plates were chosen to reduce the variability observed from sample handling in 96-well plates. 24 hours after transfection, one plate was placed in the hypoxia chamber for 90 minutes, whereas the other vessel with identically treated cells was kept under normal oxygen conditions. MTT powder (Thermo Fisher Scientific, USA) was dissolved in PBS for a concentration of 5mg/mL, after which the medium was carefully aspirated from the wells and substituted with 200 μL of the respective medium supplemented with 20 μL of the MTT solution. Cells were incubated for 4 hours at 37°C, then washed with PBS while taking great care not to move the formazan in the wells. 100 μL of dimethyl sulfoxide (Sigma Aldrich, USA) was added to the wells and, with mild mixing, incubated for 15 minutes at 37°C in an Incu-Shaker™ (Sayreville, NJ, USA). Imaging was performed on the Tecan Spark reader (Morrisville, NC, USA) at 570 nm. A flowchart of the steps is depicted in **Supplemental Figure S2**.

Scratch wound healing assay

pCPCs were cultured in 24-well plates (Greiner Bio-One International GmbH, Kremsmuenster, Austria). Reference scratches were created on the outside of the plates prior to plating of cells. The cells were transfected with the appropriate SC siRNA or a siRNA targeting circ-C12orf29 as described above. At 12 hours following transfection, scratches were created in the cell layer using a sterile 200 µl pipette tip (Biozym, Vienna, Austria). The cells were then washed with PBS and the full medium replaced with medium containing 5% FBS Superior (Sigma-Aldrich, Germany). Subsequently, the plates were either cultured under normal conditions or placed into the hypoxia chamber for 90 minutes as described above. In plates subjected to hypoxia, the medium was replenished with fresh medium containing 5% FBS. Images of the scratch in proximity to the reference lines were taken at baseline, as well as after 90 minutes and 12 hours using the Invitrogen™ EVOS™ XL Core Imaging Station (Thermo Fisher Scientific, Vienna, Austria).

SUPPLEMENTAL FIGURES

Supplemental Figure S1. Flowchart depicting the workflow of the hypoxia assay.

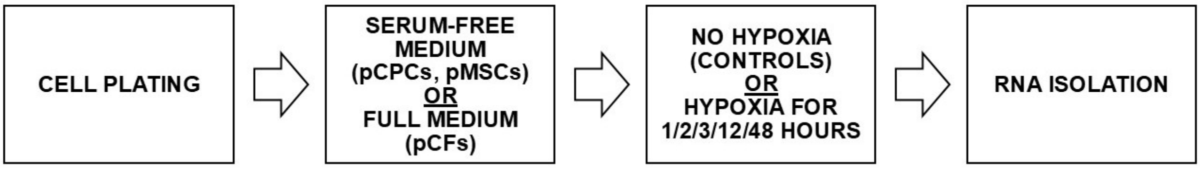

Supplemental Figure S2. Flowchart depicting the workflow of the MTT assay.

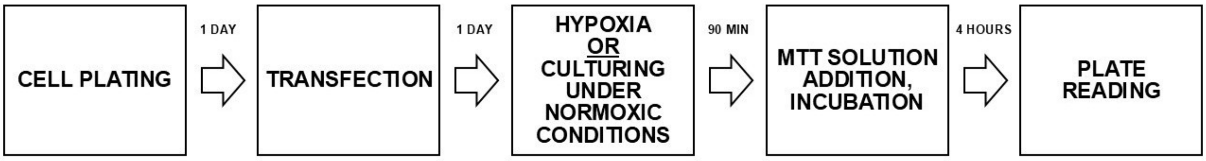

Supplemental Figure S3. siRNA-mediated knockdown of circ-RCAN2 and circ-C12orf29. Expression of circ-RCAN2 and circ-C12orf29 and their linear counterparts RCAN2 and C5H12orf29 after transfection with the siRNA targeting the respective circRNA and appropriate SC siRNA in pCPCs, pMSCs and pCFs. The change in expression is illustrated on a logarithmic scale to base 2. The level of significance is illustrated as *p=0.01-0.05, **p=0.001-0.01, ***p=0.0001-0.001. Abbreviations: porcine cardiac progenitor cells (pCPCs), porcine mesenchymal stem cells (pMSCs), porcine cardiac fibroblasts (pCFs), small interfering RNA (siRNA), Sus scrofa chromosome 5 C12orf29 homolog (C5H12orf29), Sus scrofa regulator of calcineurin 2 (RCAN2).

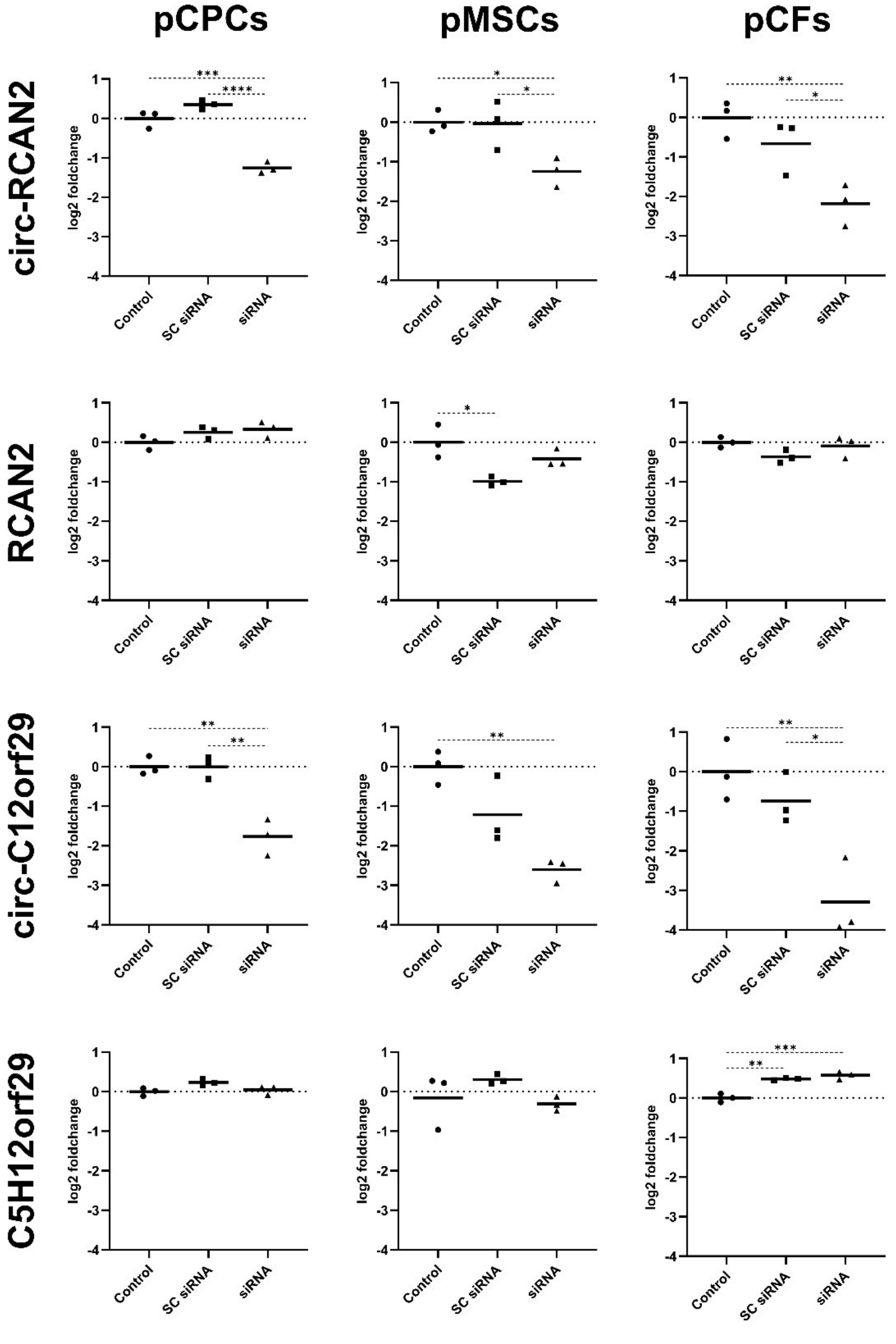

Supplemental Figure S4. Agarose gels used for Figure 2 in the main manuscript.

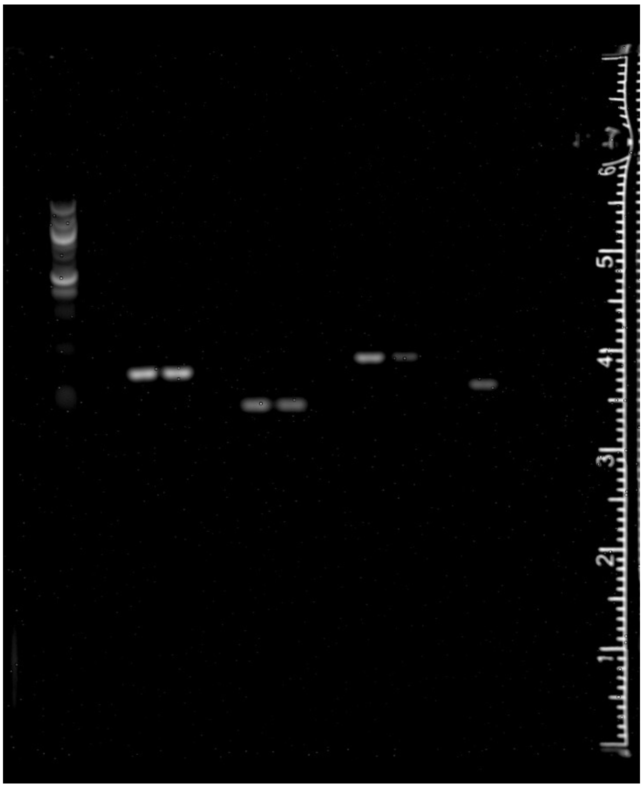

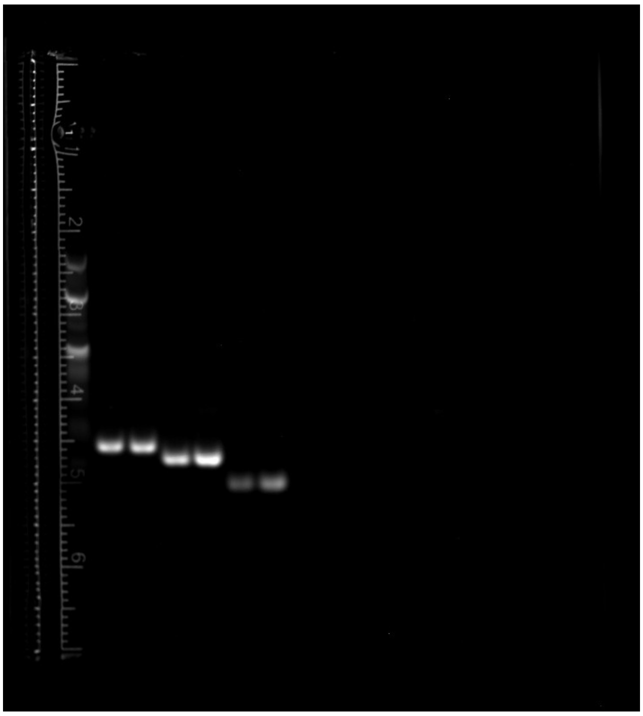

SUPPLEMENTAL TABLES

Supplemental Table S1. HIF1α, CDR1as, circ-RCAN2 and circ-C12orf29 expression after progressively longer intervals of hypoxia.

**(A) pCPCs**	**HIF1α**	**CDR1as**	**circ-RCAN2**	**circ-C12orf29**
Controls	0.00±0.08	0.00±0.19	0.00±0.20	0.00±0.13
Serum-free	0.28±0.11	1.01±0.16	0.33±0.23	0.32±0.19
Hypoxia 1h	0.36±0.08	1.32±0.24	0.34±0.27	0.48±0.08
Hypoxia 2h	0.48±0.08	1.21±0.19	0.20±0.23	0.48±0.18
Hypoxia 3h	0.47±0.10	1.16±0.22	0.14±0.31	0.29±0.28
Hypoxia 12h	0.77±0.08	1.40±0.19	0.52±0.10	0.42±0.20
Hypoxia 48h	0.54±0.08	2.44±0.21	1.18±0.32	0.66±0.21
P-Value	**<0.001**	**<0.001**	**<0.001**	**<0.001**
**(B) pMSCs**	**HIF1α**	**CDR1as**	**circ-RCAN2**	**circ-C12orf29**
Controls	0.00±0.14	0.06±0.39	0.00±0.26	0.00±0.18
Serum-free	-0.66±0.18	0.41±0.33	0.55±0.35	0.56±0.27
Hypoxia 1h	-1.30±0.09	-1.57±0.52	-0.72±0.40	-0.55±0.50
Hypoxia 2h	-0.92±0.62	-1.97±1.24	-1.12±1.28	-0.43±0.11
Hypoxia 3h	0.24±0.19	0.20±1.04	1.35±0.59	0.96±0.39
Hypoxia 12h	0.60±0.19	0.00±0.46	1.93±0.47	1.09±0.43
Hypoxia 48h	0.61±0.26	0.63±0.79	4.09±0.40	3.90±0.79
P-Value	**<0.001**	**<0.001**	**<0.001**	**<0.001**
**(C) pCFs**	**HIF1α**	**CDR1as**	**circ-RCAN2**	**circ-C12orf29**
Control	0.00±0.17	0.00±0.22	-0.02±0.71	-0.02±0.68
Hypoxia 1h	0.47±0.18	0.73±0.37	0.94±0.63	0.52±0.76
Hypoxia 2h	0.26±0.17	0.37±0.76	0.13±0.70	0.11±0.93
Hypoxia 3h	0.30±0.08	-0.03±0.87	0.13±1.59	0.26±0.99
Hypoxia 12h	0.35±0.20	-0.22±0.42	0.66±1.06	-0.32±0.62
Hypoxia 48h	0.34±0.33	-0.11±0.51	0.40±0.72	0.92±0.50
P-Value	**0.001**	**0.018**	0.404	0.078

Values are listed for (A) pCPCs, (B) pMSCs, (C) pCFs. The change in expression is shown on a logarithmic scale to base 2. Values are shown as mean and standard deviation. Significant p-values are indicated in bold. Abbreviations: porcine cardiac progenitor cells (pCPCs), porcine mesenchymal stem cells (pMSCs), porcine cardiac fibroblasts (pCFs), hypoxia inducible factor 1 alpha (HIF1α).

SUPPLEMENTAL REFERENCES

1. Zlabinger, K. *et al.* MiR-21, MiR-29a, GATA4, and MEF2c Expression Changes in Endothelin-1 and Angiotensin II Cardiac Hypertrophy Stimulated Isl-1+Sca-1+c-kit+ Porcine Cardiac Progenitor Cells In Vitro. *Cells* **8**, 1416 (2019).
2. Zlabinger, K. *et al.* Matrix Metalloproteinase-2 Impairs Homing of Intracoronary Delivered Mesenchymal Stem Cells in a Porcine Reperfused Myocardial Infarction: Comparison With Intramyocardial Cell Delivery. *Front Bioeng Biotechnol* **6**, 35 (2018).
3. Elbashir, S. M., Martinez, J., Patkaniowska, A., Lendeckel, W. & Tuschl, T. Functional anatomy of siRNAs for mediating efficient RNAi in Drosophila melanogaster embryo lysate. *EMBO J* **20**, 6877–6888 (2001).
4. Ui-Tei, K. *et al.* Guidelines for the selection of highly effective siRNA sequences for mammalian and chick RNA interference. *Nucleic Acids Res* **32**, 936–948 (2004).
5. Altschul, S. F., Gish, W., Miller, W., Myers, E. W. & Lipman, D. J. Basic local alignment search tool. *J Mol Biol* **215**, 403–410 (1990).
6. Ye, J. *et al.* Primer-BLAST: A tool to design target-specific primers for polymerase chain reaction. *BMC Bioinformatics* **13**, 134 (2012).
